# CryoEM Workflow Acceleration with Feret Signatures

**DOI:** 10.3390/ijms25147593

**Published:** 2024-07-11

**Authors:** Pierre Nottelet, Peter Van Blerkom, Xiao-Ping Xu, Dorit Hanein, Niels Volkmann

**Affiliations:** 1 Department of Chemistry and Biochemistry, University of California Santa Barbara, Santa Barbara, CA 93106, USA; 2 The Scintillon Institute, La Jolla, San Diego, CA 92121, USA; 3 Department of Biological Engineering, University of California Santa Barbara, Santa Barbara, CA 93106, USA; 4 Department of Electrical and Computer Engineering, University of California Santa Barbara, Santa Barbara, CA 93106, USA

**Keywords:** cryogenic electron microscopy, data processing, workflow optimization, structure determination, structural biology, analytical tool, single particle analysis, Feret signature

## Abstract

Common challenges in cryogenic electron microscopy, such as orientation bias, conformational diversity, and 3D misclassification, complicate single particle analysis and lead to significant resource expenditure. We previously introduced an in silico method using the maximum Feret diameter distribution, the Feret signature, to characterize sample heterogeneity of disc-shaped samples. Here, we expanded the Feret signature methodology to identify preferred orientations of samples containing arbitrary shapes with only about 1000 particles required. This method enables real-time adjustments of data acquisition parameters for optimizing data collection strategies or aiding in decisions to discontinue ineffective imaging sessions. Beyond detecting preferred orientations, the Feret signature approach can serve as an early-warning system for inconsistencies in classification during initial image processing steps, a capability that allows for strategic adjustments in data processing. These features establish the Feret signature as a valuable auxiliary tool in the context of single particle analysis, significantly accelerating the structure determination process.

## 1. Introduction

The advancement of cryogenic electron microscopy (cryoEM) has revolutionized structural biology by enabling the high-resolution visualization of complex macromolecules [1,2,3]. However, the full potential of this technology is often hindered by the need for efficient sample screening and preparation [4,5,6]. Streamlining these preliminary stages is essential to rapidly progress to data collection, saving valuable time and resources. Experimental challenges associated with sample preparation require fast assessment to optimize data quality and minimize the resources spent.

A significant experimental challenge in cryoEM is the issue of preferred orientation, which is a non-trivial problem consuming extensive microscope time and computational resources during screening [7,8,9]. This issue arises as particles near the air–water interface are subjected to surface tension, leading them to orient preferentially towards the interface, and in more severe cases, causing denaturation [10,11]. Typically, such orientation preferences are detected only after comprehensive data analysis, by which time substantial efforts and resources may have already been expended. Although tomography can provide clues for identifying preferred orientation, these indicators are often inconclusive, offering no reliable method to address this issue during the early screening or data collection stages [10].

Recently, we introduced an in silico method for the rapid evaluation of the homogeneity of samples featuring particles with simple shapes, such as nanodiscs [12]. This method analyzes the Feret diameter distribution (Feret signature) derived from electron microscopy particle projections. We have now broadened the scope of the methodology to enable the real-time detection of preferred orientations during data collection. Our approach requires only a limited number of particles (as few as 1000 per experimental dataset) and thus can streamline the data collection workflow, empowering researchers to modify acquisition parameters midstream. This adaptive capability allows for the strategic adjustment of data collection, such as shifting to tilted data collection or discontinuing unfruitful sample imaging altogether. In addition to the ability to detect preferred orientations during the screening phase, we demonstrate that the Feret signature approach can serve as an early-warning system to identify inconsistencies with classification, providing a preemptive tool for refinement strategy adjustments. These properties position the Feret signature approach as a valuable supplementary tool to single particle analysis (SPA) for the acceleration of the structure determination process.

## 2. Results

The primary objective of this study is to demonstrate the effectiveness of the Feret signature method in addressing common challenges in cryoEM, such as orientation bias, conformational diversity, and inconsistencies in classification. We present evidence that Feret signature protocols are not only quick and efficient but also robust and valuable in assessing these types of inconsistencies.

Thus, employing the Feret signature method could potentially accelerate the evaluation of sample readiness and the interpretation of cryoEM data. The approach is useful for assessing both the quality of disc-shaped sample preparation [12] and, as shown here, the characteristics of sample orientations and conformation variability of arbitrary samples. Integration of the Feret methodology into data collection and analysis workflows could lead to faster and cheaper data acquisition trials, more reliable data interpretation, and ultimately more accurate structural models.

The Feret signature refers to the distribution of the maximum Feret diameters (*F_max_*) of particles within the SPA datasets [12]. This distribution encapsulates information about shape diversity and particle orientation. Analyzing and comparing the Feret signatures of data recorded at different tilt angles can provide insights about potential preferred orientation. Comparing Feret signatures from experimental data with simulated data can also provide information about potential heterogeneity, particle picking, or inconsistencies in classification if the distributions are significantly different. However, in contrast to the Feret analysis targeting preferred orientation, this aspect requires one or more reference structures.

### 2.1. Assessment of Preferred Orientation 

#### 2.1.1. Data Displaying Strong Preferred Orientations

We utilized an experimental dataset of Influenza Hemagglutinin Trimer [8] to demonstrate the capability of Feret signatures in detecting preferred orientation. The analysis of a standard dataset (collected at 0° tilt angle) revealed that the majority of the particles were oriented with their long axis perpendicular to the air–water interface and that side views were almost entirely missing. The authors then tilted the sample by 40° to increase the orientation coverage [13] and succeeded in obtaining a high-resolution reconstruction [8]. Indeed, tilting by 40° significantly increased the *F_max_* range coverage of particle orientations. A comparison of the Feret signatures calculated from the 0° tilt data and 40° tilt data shows very clear differences and is a robust indicator for the extensive preferred orientation issue.

**Figure 1:**
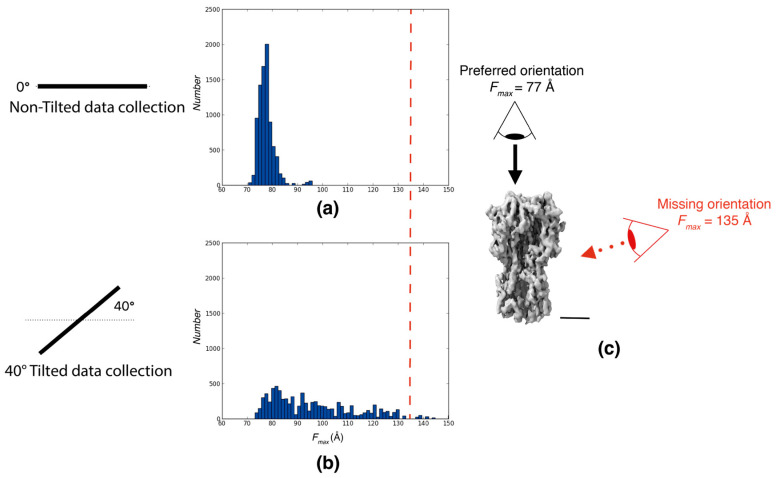
An assessment of preferred orientation using Feret signatures. The *F_max_* distributions of particles randomly picked from particle extracts of 0° tilt and a tilted dataset of the Influenza hemagglutinin trimer from Tan et al. (2017) [8]. (**a**) *F_max_* distributions from particles picked from the 0° tilt dataset. (**b**) *F_max_* distributions from particles picked from the 40° tilt dataset. (**c**) Influenza hemagglutinin trimer reconstruction at 4.2 Å resolution (EMD:8731; scale bar = 40 Å). For all panels, black dots indicate the *F_max_* values for the preferred orientation, and red dots indicate the *F_max_* values for the missing orientation. The red line in (**a**,**b**) corresponds to the view marked in red in (**c**).

**Figure A1:**
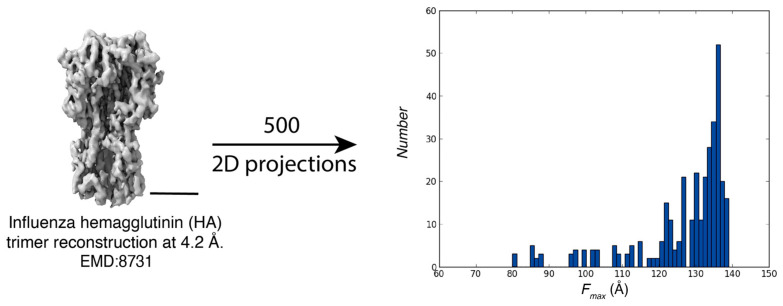
Characterization of preferred orientations using simulated Feret signatures. *F_max_* distributions from 2D projections of influenza hemagglutinin trimer reconstruction at 4.2 Å resolution (EMD:8731; scale bar = 40 Å).

The 0° tilt dataset exhibited a very constrained *F_max_* distribution, ranging only between 70 and 95 Å with a mode of the distribution at 80 Å. This narrow range is indicative of a pronounced preferred orientation within this sample (Figure 1a). In contrast, the 40° tilt dataset displayed a broader and more diverse range of *F_max_* between 70 and 150 Å. A comparison with simulated data using the refined final structure shows that the covered range between the tilted data and the simulation closely matches even though the relative frequencies of the individual orientations are different (Figure A1). This discrepancy allows the definition of a quantifiable measure for detecting severe cases of preferred orientations: If the range covered by the Feret signatures in untilted images is significantly lower than the range covered in tilted images, preferred orientation is likely. This measure is simple to calculate and can be used as part of an initial automated screening procedure.

#### 2.1.2. Data with Good Coverage of Orientations

**Figure 2:**
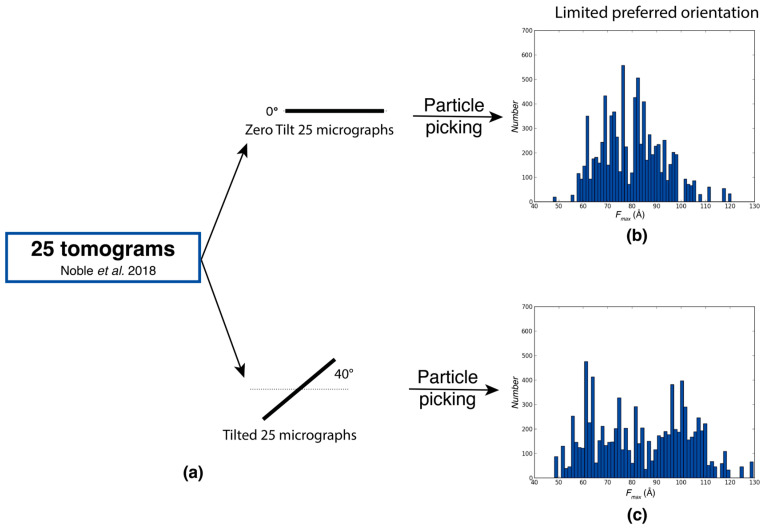
Assessment of limited preferred orientations using Feret signatures. *F_max_* distributions of particles randomly picked from 0° tilt dataset with limited preferred orientation, and tilted dataset of Rabbit Muscle Aldolase from Noble et al. (2018) [10]. (**a**) Extraction of micrographs and particle picking from tilt series at 0° tilt and 40° tilt. (**b**) *F_max_* distributions from particles selected from 0° tilt micrographs. (**c**) *F_max_* distributions from particles selected from 40° tilt micrographs.

In the evaluation of the experimental Rabbit Muscle Aldolase data [10] extracted from tilt images at different angles from 25 tomograms, analyses of *F_max_* distributions under 0° tilt and 40° tilt conditions revealed similar profiles, with an *F_max_* distribution between 60 and 120 Å for both cases (Figure 2b). High-resolution SPA analysis showed that preferred orientations in aldolase data are nearly absent with only a slight preference for views along the long axis [14]. This preference is reflected in the *F_max_* distributions where a somewhat higher population of larger *F_max_* values is observed for the tilted data (Figure 2a). The comparison of tilted and untilted data provides a robust measure for assessing the extent of preferred orientations in the sample. If the covered ranges of tilted and untilted data are the same and shifts in the relative frequencies are minor, untilted data are sufficient for high-resolution structure determination. If the range is severely restricted in the untilted data with respect to the tilted data, extensive preferred orientation is present in the sample.

#### 2.1.3. Feret Signature Robustness: Analysis of Randomly Split Particle Datasets

**Figure A2:**
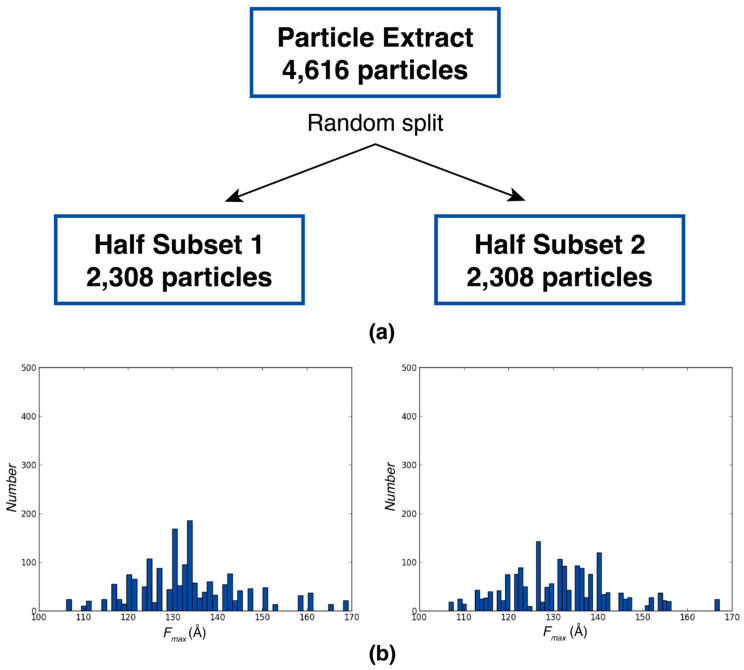
Robustness of Feret signatures to random sampling. (**a**) Schematic of random split into two equivalent subsets. Initial dataset is beta-galactosidase extract from Relion tutorial. (**b**) *F_max_* distributions from particles randomly selected from first subset (**left panel**) and second subset (**right panel**).

The robustness of the Feret signature method was further validated through the analysis of the beta-galactosidase particles extracted from the Relion tutorial dataset (beta-galactosidase (Sjors H.W. Scheres Relion tutorial dataset (v4.0)). This dataset was randomly split into two equal subsets. The *F_max_* distributions remained consistent across both subsets, with *F_max_* distributions within the 110 and 160 Å range. This similarity highlights the reproducibility and reliability of Feret signature extraction even with smaller particle subsets (2308 particles in each subset) (Figure A2). This consistency not only affirms the reproducibility of the Feret signature extraction process but also demonstrates its reliability when applied to smaller subsets of particles. The well-characterized nature of the beta-galactosidase dataset served as an ideal test case, providing ample information about the sample that further reinforced the reliability of our approach.

#### 2.1.4. Minimal Number of Particles Required for Preferred Orientation Detection 

**Figure 3:**
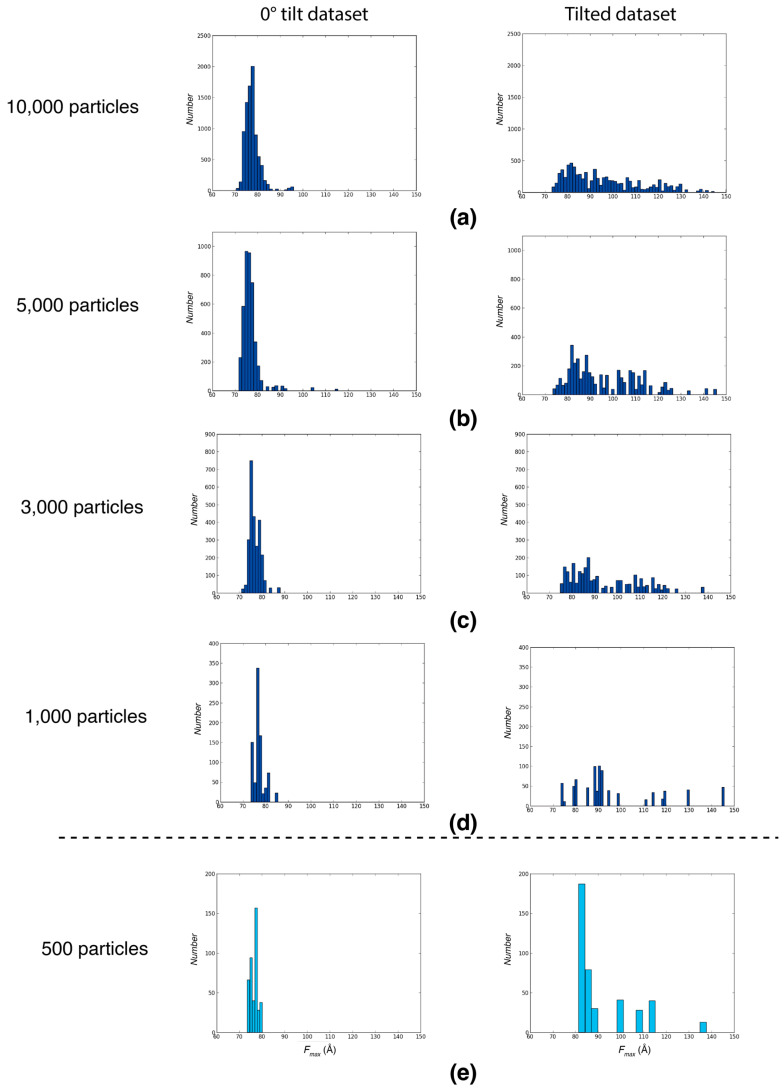
Feret signature robustness during preferred orientation assessment. The *F_ma_*_x_ distributions of particles randomly picked from a 0° tilt dataset with preferred orientation (**left panels**) and the respective tilted dataset (**right panels**) from Tan et al. (2017) [7], with the same dataset used for Figure 1. (**a**) *F_max_* distributions from 10,000 randomly picked particles per tilt. (**b**) *F_max_* distributions from 5000 randomly picked particles per tilt. (**c**) *F_max_* distributions from 3000 randomly picked particles per tilt. (**d**) *F_max_* distributions from 1000 randomly picked particles per tilt; the minimum number of particles required to detect a Feret signature. (**e**) *F_max_* distributions from 500 randomly picked particles per tilt. A detailed analysis of the influence of noise using simulation studies of differently sized and shaped particles [12] shows that the distinction between different Feret signatures is robust even if the signal-to-noise ratio is low. However, if the particles under investigation are nearly globular, the expected differences between the tilted and untilted distributions will be more subtle and a higher signal-to-noise ratio (i.e., more particles) may be necessary to make a robust conclusion about preferred orientations. Nevertheless, the number of required images will still be small compared to high-resolution datasets.

To determine the minimum number of particles necessary for the reliable determination of Feret signatures, we successively reduced the number of particles for the analysis and compared the resulting Feret signatures to that of the entire dataset. For both 0° tilt and tilted datasets of Influenza hemagglutinin trimers, reliable signatures were detectable with as few as 1000 particles per tilt, with similar *F_max_* spread and similar distribution shape in comparison to the full dataset. Once the number of particles becomes too low (for example, 500), the overall distribution shape and range cannot be reliably retrieved by random sampling (Figure 3). 

The small number of particles required for Feret signature detection is advantageous in the early stage of cryoEM workflows. It facilitates early insights into critical sample characteristics, such as preferred orientation, thereby enabling efficient preliminary screenings with limited data, such as the data collection of 10 to 30 tilted and untilted micrographs in the screening step which would be sufficient to test for preferred orientation and detect it if it is present. Traditionally, preferred orientation cannot be easily detected during data collection and often only becomes apparent after extensive processing which is not only time-intensive but also demanding in terms of computational and human resources. By incorporating the Feret signature analysis early in the process, preferred orientation can be detected during the screening step, enabling more informed decisions on whether to proceed with tilted data collection or allocate resources to generate alternative samples.

### 2.2. Detection of Classification Inconsistencies with Feret Signatures 

Previously, Xu et al. (2016) [15] identified four conformations of full-length αIIbβ3 integrin at a low resolution. To potentially retrieve higher-resolution information, we collected a dataset significantly closer to focus than the original data. We used this dataset to demonstrate the ability of Feret signatures to detect classification inconsistencies in datasets with high heterogeneity and low signal-to-noise ratios. By comparing Feret signatures of classified particles with simulated ones and examining these distributions across different classification steps, we were able to pinpoint inconsistencies early in image processing, particularly highlighting major challenges with the classification of the closer-to-focus data. We used two different classification steps to perform this analysis. (1) Classes derived from particle picking with the different low-resolution structures as templates and (2) classification based on 3D refinement within cryoSPARC using the original four low-resolution structures as starting points.

**Figure 4:**
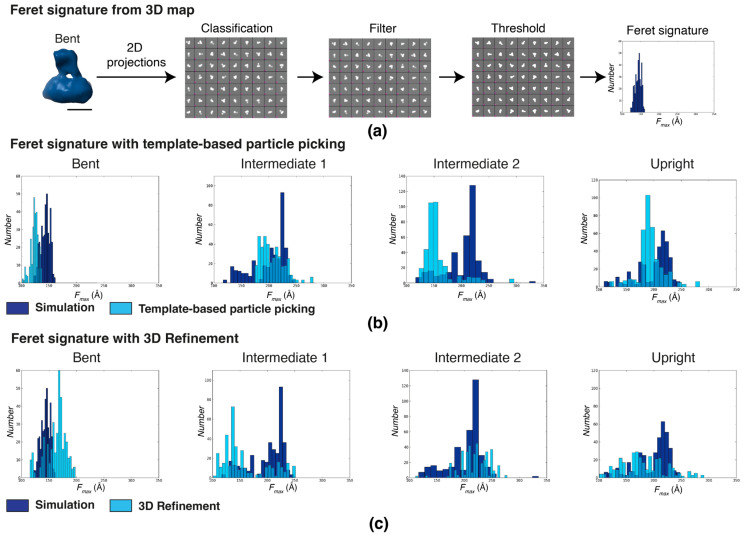
Feret signatures identify inconsistency in classification during the early stages of image processing. *F_max_* distributions of particles randomly picked using low-resolution templates of the four conformations determined by Xu et al. (2016) [15] (scale bar = 60 Å). (**a**) The procedure to generate the simulated Feret signature from synthetic 2D projections of a cryoEM template and (**b**) *F_max_* distributions of the simulated Feret signatures from the four integrin conformations: bent, intermediate 1, intermediate 2, and upright (from left to right, represented in dark blue). These are superimposed with the corresponding experimental *F_max_* distributions (light blue) derived by template-based particle picking. (**c**) Simulated *F_max_* distributions from the four integrin conformations (dark blue), superimposed with the corresponding experimental *F_max_* distributions derived from particles classified by 3D refinement.

The Feret signatures derived from simulations of the four previously determined integrin conformations (bent, intermediate 1, intermediate 2, and upright) were compared with experimental data (Figure 4). Simulated Feret signatures were obtained from 500 2D noise-free projections with full angular coverage for each full-length integrin conformation (Figure 4a). For the bent conformation, the simulated data displayed a Feret signature spread between 120 and 160 Å with a well-defined peak at 145 Å. For the intermediate and upright conformations, the Feret signature spreads between 120 and 250 Å. The intermediate 1 conformation displays a bimodal distribution with the main peak at 220 Å and a secondary peak at 140 Å. The shape of the intermediate 2 distribution is quite similar with the main peak also at 220 Å and a secondary peak at a somewhat higher *F_max_*, around 145 Å. The simulated Feret signature of the upright conformation has a more even spread and only displays a peak at 220 Å.

For the bent conformation, the Feret signature of particles is classified based on the template-matching spread between 100 and 140 Å with a peak at about 120 Å, essentially a shift in the distribution towards a smaller *F_max_* by 20 Å. Classification by 3D refinement results in a Feret signature spread of 130 to 190 Å with a single peak at 160 Å, displaying a slight skew rather than the symmetrical distributions of the simulated and template-based distributions. While the shift in the template-based distribution could be explained by slight offsets in thresholds or defocus, the skewness and broadening of the 3D-refinement-based distribution indicates contamination from the more extended conformations.

For the intermediate 1 conformation, the template-based distribution spreads between 175 and 275 with a single broad peak at about 190 Å, indicating a lack of orientations at low *F_max_* values as well as the absence of some orientations at higher *F_max_* values. After 3D refinement, the behavior completely changes, now displaying a single peak at 140 Å and a highly diminished population at larger *F_max_* values. For the intermediate 2 conformation, the template-based distribution mimics the distribution of the bent conformation, indicating significant contamination with particles from the bent conformation. After 3D refinement, the distribution again changes significantly, now more closely resembling the simulated distribution but with a much-diminished peak. For the upright conformation, the template-based conformation is similar to the simulated one but, just like the bent conformation, with the distribution shifted by about 20 Å towards smaller *F_max_* values. After 3D refinement, the distribution matches quite well at smaller *F_max_* values but lost contributors at larger *F_max_* values.

Together, the observed shifts in the distributions indicate a high degree of instability during classification. Consistent with our previous analysis that indicated higher conformational variability in the intermediate conformations [15], the most pronounced inconsistencies are apparent in the intermediate conformation with the Feret signature analysis. While the changes in the distributions of the bent and upright conformations are somewhat less pronounced, the changes are still apparent, especially after 3D refinement. The observed classification instability indicates an inability to accurately determine the correct class for the particles, even if a reference is provided. The most likely cause for this behavior is that the signal under these imaging conditions is insufficient for alignment and classification. The conclusion is that the defocus range for this data collection was too close to focus and should be increased to stabilize classification behavior. Because we are looking at a shift in distributions between different classification schemes, using exactly the same particle set, the conclusion is robust even for low signal-to-noise data. 

The extraction of the Feret signature (*F_max_*) is inherently low-resolution and more tolerant regarding classification and alignment accuracy than what is required for high-resolution structure determination. It is designed to be run during initial screening before high-resolution data collection. However, the method may not always succeed in extracting meaningful Feret signatures from a given dataset with a given set of parameters. Factors like particle size, signal-to-noise ratio, chosen defocus, tendency to aggregate, or conformational variability can all adversely affect the accuracy of *F_max_* extraction. Although it is not possible to determine in advance if the procedure will be successful, the shape of the Feret signature distribution can indicate the success of the analysis in real time during the screening phase. Random class assignments and alignments will result in a Gaussian distribution that is invariant to sub-classification and data collection at different tilt angles. If this occurs, remedies include increasing contrast by adjusting defocus, an electron dose, collecting additional data, or using a Phase Plate device if available.

The potential of Feret signatures to detect inconsistencies in classification at an early stage in data processing can be particularly valuable for datasets comprising a large set of multiple conformations, where such issues might otherwise go unnoticed until advanced stages of analysis. Traditional SPA techniques, including 3D classifications, may offer insights into misclassification but often only at a later stage and with complex interpretation challenges. 

## 3. Discussion

This study highlights the efficacy of the Feret signature method in tackling prevalent challenges in cryoEM such as orientation bias, conformational diversity, and inconsistencies in classification. Integrating this technique into existing workflows can streamline data collection and analysis, reducing costs and improving the reliability of structural models. The method not only speeds up the assessment of sample readiness and enhances the accuracy of cryoEM data interpretation, but it also ensures robust sample evaluation.

The Feret signature is based on the distribution of maximum Feret diameters (*F_max_*) within SPA datasets, capturing essential data on shape diversity and particle orientation. By analyzing Feret signatures at different tilt angles, researchers can detect potential preferred orientations. Furthermore, comparisons between experimental and simulated data using Feret signatures identify inconsistencies in classification, highlighting its broad applicability in refining SPA strategies.

Here, we selected a sampler of datasets to exemplify the utility of the new capabilities of the extended Feret signature that include the methods below.

The Influenza Hemagglutinin Trimer dataset demonstrates how Feret signatures can detect preferred orientations. This dataset was also used to estimate the minimum number of particles required to obtain reliable Feret signatures for the detection of preferred orientations. The Rabbit Muscle Aldolase dataset was chosen for its low degree of preferred orientations, confirming the consistency of the Feret signature assessment of preferred orientations. The analysis also verifies that only a few images (<30) are required to obtain a robust Feret signature signal even for very noisy data (the images were extracted from tomographic tilt series with an electron dose of 1.54 e^−^/Å^2^ for 0° tilt and 1.84 e^−^/Å^2^ for 40° tilt.). An application of the Feret signature method to experimental and simulated full-length Integrin datasets revealed potential inconsistencies in classification. Lastly, the beta-galactosidase dataset from the Relion tutorial highlighted the reliability and reproducibility of Feret signatures, even with limited data, with similar results across randomly split subsets of the same dataset.

Our analysis suggests opportunities for the inclusion of the Feret signature methodology into data collection workflows to increase productivity. At the screening stage, the collection of 20–30 images at 0° and 40° tilt, preferably at high defocus to increase contrast, would allow for the evaluation of the presence of preferred orientations in the sample before high-resolution data collection commences. Because extraction of *F_max_* does not require high-resolution detail, much higher doses and defoci than those applied during high-resolution data collection can be used. Employing the Feret signature analysis in this way would allow for an adjustment of data collection strategies (e.g., tilted data collection) or aborting data collection altogether aiming to improve the orientation distribution by other means. During particle picking and initial 3D refinement in the presence of multiple conformations, the comparison between Feret signatures of the picked or classified subset and the reconstruction of the corresponding class can serve as an early detection system for potential issues allowing us to steer the data processing strategy at the early stages of the refinement process.

## 4. Materials and Methods

### 4.1. Materials

#### 4.1.1. CryoEM Data 

Full-length integrin: Vitrified cryoEM grids (Quantifoil R 1.2/1.3 holey carbon film on a 400 mesh) of full-length, membrane-embedded human αIIbβ3 integrin complexes were generated by Xu et al. (2016) [15]. Data were recorded at the Caltech cryoEM facility, on a Titan Krios G3i (Thermo Fisher Scientific, Waltham, MA, USA) operated at 300 kV equipped with a K3 summit camera and a Gatan Imaging Filter (GIF) at 10 eV, operated at ×105,000 magnification and a nominal pixel size of 0.832 Å using SerialEM (v4.0.13) [16]. Movies were collected across a defocus range of −0.5 µm to −1.7 µm and with a dose of 1.21 electrons per Å^2^ per frame. A total of 4984 movies were recorded with 40 frames each. These movies were corrected for beam-induced motion using Patch Motion Correction, and CTF (contrast transfer function) parameters were assessed using the computer program Patch CTF Estimation from CryoSPARC (v4.4.0) [17]. Particles were picked from the corrected micrographs, using projections of the four integrin conformation (bent, intermediate 1, intermediate 2, and upright) maps from Xu et al. (2016) [15] as low-resolution templates (between 30 and 45 Å resolution). A total of 3,112,128 particles were picked, consisting of 1,031,720 bent, 836,285 intermediate 1, 726,364 intermediate 2, and 517,759 upright. Particles picked with the bent conformation template were extracted using a box size of 360 pixels binned to 180 pixels. For the intermediate 1 and intermediate 2 conformations, particles were extracted using a box size of 540 pixels binned to 270 pixels. For the upright conformation, particles were extracted using a box size of 720 pixels binned to 360 pixels. Particles for each picked subset were classified into 500 classes using the reference-free 2D classification implemented in CryoSPARC, to improve the overall quality of the picked subset by removing classes with spurious density or an excessive amount of noise.

Particles selected from the reference-free 2D classification were merged, and duplicates were removed using the “Remove Duplicate Particles” tool implemented in CryoSPARC, with a minimum separation distance of 20 Å, resulting in a total of 22,215,099 particles. These particles were then 3D classified into four subsets based on four conformations with the 3D “Heterogeneous Refinement” tool implemented in CryoSPARC, using the previously determined low-resolution structures of the four integrin conformations from Xu et al. (2016) [15]. The four final subsets were composed of 662,927 bent, 691,278 intermediate 1, 379,401 intermediate 2, and 28,094 upright particles. Each subset was further classified into 500 classes using the reference-free 2D classification implemented in CryoSPARC to improve the overall quality of the picked subset by removing classes with spurious density or an excessive amount of noise.

Influenza Hemagglutinin Trimer: Two particle datasets were downloaded from the EMPIAR database. These data were generated using a Titan Krios (Thermo Fisher Scientific) operated at 300 kV equipped with a K2 summit camera by Tan et al., 2017 with 466 micrographs without tilt (EMPIAR-10096) and 847 at a 40° tilt angle (EMPIAR 10097) as described by Tan et al. (2017) [8]. A total of 130,000 particles for each dataset, as extracted and deposited by Tan et al. (2017), were used in this study.

Rabbit Muscle Aldolase: In total, 25 tilt-series were downloaded from the EMPIAR database (EMPIAR-10130). These tilt series were collected by Noble et al. (2018) [10] on a Titan Krios (Thermo Fisher Scientific) operated at 300 kV equipped with a K2 summit camera. Tilt-series were collected bi-directionally with a range of −45° to 45° and a 3° increment as described by Noble et al. (2018) [10]. Micrographs at 0° tilt and 40° tilt from each tilt series were used to pick 13,892 and 17,821 particles from the 0° and 40° tilts, respectively, using the blob picker for CryoSPARC [17]. These particles were 2D classified and were further analyzed as described.

Beta-galactosidase: A dataset provided by Takayuki Kato collected on a JEOL (Tokyo, Japan) CRYO ARM 200 microscope (EMPIAR-10204) with a pixel size of 1.24 Å was used as a test dataset in the Relion tutorial by Sjors H.W. Scheres (v4.0). We used in this study the 4748 particles that were extracted in a 360-pixel box size and are provided with the Relion tutorial. 

#### 4.1.2. Feret Signatures: Simulated Feret Signatures from 3D Maps

For the integrin dataset, the 3D reconstructions of the four integrin conformations (bent, intermediate 1, intermediate 2, and upright) from Xu et al. (2016) [15] were utilized to produce 500 2D projections. For the Influenza Hemagglutinin Trimer dataset, a 3D reconstruction at a resolution of 4.2 Å, derived from particles imaged at a 40° tilt as reported by Tan et al. (2017) (EMDB-8731) [8], was used to generate 500 2D projections. Feret signatures were determined for both simulated datasets through a multi-step procedure typical of Feret signature analysis [12]. However, unlike the standard method which uses 50 projections per class as a default, reference-free classification and averaging were performed using only five simulated projections per class because of the absence of noise.

#### 4.1.3. Feret Analysis 

##### Characterization of Four Integrin Conformation Distributions

For the analysis of template-based particle picking performance, Feret signatures were calculated from experimental data after template-based particle picking using the previously determined four conformations [15] followed by cleanup based on reference-free classification. For the analysis of potential classification issues during 3D refinement, Feret signatures were calculated from particles extracted from the four classes obtained after 3D refinement. Simulated Feret signatures were obtained, as previously described, from 3D reconstructions of the four integrin conformations. 

##### Characterization of Influenza Hemagglutinin Trimer Distributions

Feret signature distributions for both 0° tilt and 40° tilt datasets of Influenza Hemagglutinin Trimer were analyzed using particle stacks from Tan et al. (2017) (EMPIAR-10096 and EMPIAR-10097, respectively) [8]. From each dataset comprising 130,000 particles, *F_max_* signatures were determined from samples ranging from 500 to 10,000 randomly selected particles.

##### Characterization of Rabbit Muscle Aldolase Distributions from Tilt Series

For the Rabbit Muscle Aldolase, 25 micrographs at 0° tilt and 25 micrographs at 40° tilt were extracted from the tilt series provided by Noble et al. (2018) [10] (EMPIAR-10130). Particle picking was conducted in CryoSPARC v4.4.0 [17] using the blob picker, targeting particles with diameters between 60 Å and 120 Å. Particles were extracted using a box size of 256 pixels and subsequently 2D classified. A total of 13,417 particles from the 0° tilt and 17,811 particles from the 40° tilt were selected for analysis. *F_max_* signatures were then extracted from 10,000 randomly selected particles from each tilt subset.

##### Characterization of Beta-Galactosidase

For the beta-galactosidase analysis, 4748 particles were extracted from the Relion tutorial dataset and subjected to 2D classification to retain the best 4616 particles. These particles were then randomly divided into two equal subsets, with *F_max_* signatures extracted from 2308 particles in each subset.

### 4.2. Methods

#### 4.2.1. Data Processing and Particle Selection

For the integrin and Rabbit Muscle Aldolase datasets, initial image processing steps and particle picking were carried out as detailed in the Materials section. For the beta-galactosidase dataset, particles were extracted from the Relion tutorial test data and were used. Following particle extraction, datasets were subjected to several rounds of 2D classification in CryoSPARC to ensure that only well-defined particles were retained for further analysis.

#### 4.2.2. Feret Signature Extraction and Analysis

Feret signature analysis, described by Vilela et al. (2022) [12], was originally introduced to characterize the size and shape distributions of nanodisc samples. The specific constraints on the nanodisc shape (i.e., either round or oblate disc with fixed thickness) allow for the extraction of shape parameters from Feret diameter distributions alone [12]. Here, we extend the Feret signature analysis to particles of arbitrary shapes. We focus on the comparison of Feret signatures between different experimental conditions (tilted versus untilted) to detect preferred orientations and analyze the comparison between simulated and experimental Feret signatures to explore their usefulness for detecting inconsistencies in classification. 

**Figure A3:**
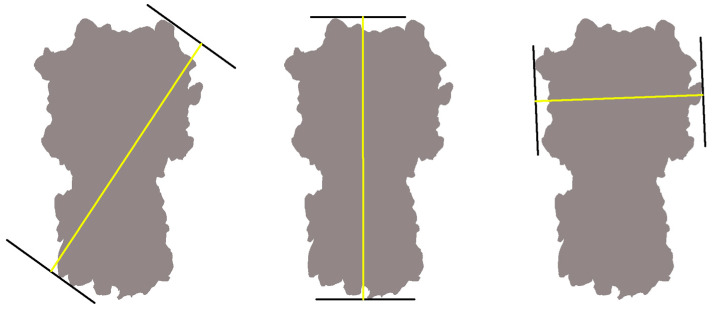
Feret diameters. A Feret diameter (yellow) is the distance between two parallel tangents (black lines) of an object (gray); here, the side-view projection of Influenza Hemagglutinin. The projection can have many different Feret diameters, three are shown here, but only the maximum (center) is kept for the analysis.

The extraction and analysis of Feret signatures were described in depth previously [12]. Briefly, a multi-step procedure is employed that includes (1) reference-free classification and averaging of picked particles to enhance the signal-to-noise ratio, (2) denoising to allow for adequate binarization, (3) entropy-based binarization, and (4) the extraction of the maximum Ferret diameters, *F_max_*, for each projection image. Step (1) and/or step (2) can be omitted if the signal-to-noise ratio is high. A Feret diameter is the distance between two parallel tangents of an object’s boundaries, similar to measuring its size with a caliper (Appendix A, Figure A3). *F_max_* is the maximum of all possible Feret diameters. The histogram of all *F_max_* values in a particle set is termed its Feret signature. Note that *F_max_* is dominated by the overall shape of the projection image, not its details, making *F_max_* robust in light of minor classification and alignment errors in case of low signal-to-noise ratios [12]. The Feret signature software is distributed as an add-on to the pyCoAn 0.3 framework (https://github.com/pyCoAn/distro), an extended Python version of the CoAn package [18].

##### Loading Image Stacks

Initially, the particles, previously selected with automated picking processes as described, are loaded into pyCoAn. In pyCoAn, particle images are managed through the “ImgStack” object. For large datasets, the analysis can be limited to a subset to preserve computational resources while maintaining Feret signature accuracy. 

##### Running Feret Signature Evaluation

The core of the methodology is the “feret_eval” function, which processes the entire stack of particle images to compute Feret signatures. This function manages a series of image processing steps including the classification, averaging, filtering, and thresholding of class averages to extract the Feret signatures.

##### Classification and Averaging

To limit the effects of noise and ensure the reliability of Feret diameter measurements, a classification step is used where the number of particles per class is optimized. This balances the detail captured against the noise level, with a default setting of 50 images per class. This classification is performed using the “feret_classes” function which is based on double autocorrelation functions [19]. The underlying algorithm is fast and works well for Feret diameter extraction which does not rely on high-resolution features. However, it does require good contrast to work efficiently and performs best for data with high defocus. In cases where the contrast is low, it is recommended to input already pre-classified 2D class averages from external packages that are better suited for low-contrast data such as those obtained from close-to-focus data. For this study, we used pre-classified 2D classes calculated and pre-screened with CryoSPARC for the Influenza Hemagglutinin Trimer and integrin datasets. 

##### Filtering and Noise Reduction

Following classification, a filtering step based on non-local means [20] is applied to reduce noise. The default value is usually sufficient, but the strength of the filtering can be adjusted as necessary to allow for better thresholding.

##### Comprehensive Analysis and Visualization

Upon completion of the filtering step, the feret_eval function finalizes the Feret signature extraction by thresholding the denoised class averages using Renyi’s entropy [21] extraction of the central segment and the determination of the maximum Feret diameter (*F_max_*) of the particle projections as described previously [12]. Visualization tools within pyCoAn are employed to monitor the results of the processing at each step.

##### Data Management and Reproducibility

To ensure reproducibility, all processed data, including the computed Feret signatures, are saved together with all metadata in a format that can be re-imported into pyCoAn. This allows for future validations or additional analyses without the need to rerun the entire processing pipeline.

##### Parameter Optimization and Continuous Refinement

While most default parameters work well for high-contrast datasets, adjustments of the parameters for each step of the Feret signature evaluation are implemented in the pipeline for more challenging cases. This iterative refinement is guided by the initial outcomes and visual assessments of the processed images, ensuring optimal data quality and interpretative accuracy.

#### 4.2.3. Simulation of Feret Signatures

Using the 3D reconstructions previously mentioned in the materials section, Feret signatures were simulated for each dataset. For the integrin complexes, 500 2D projections were generated for each conformation using the “create templates” job in CryoSPARC [17]. Similarly, for the Influenza Hemagglutinin Trimer, projections were derived from a 3D reconstruction at 4.2 Å resolution. These projections were used to compute Feret signatures using a lower number of 2D image averaging followed by similar filtering and noise reduction, thresholding, segmentation, and signature extraction parameters as described previously. 

## 5. Conclusions

The use of the Feret signature method has demonstrated remarkable fidelity in detecting and addressing critical sample issues such as preferred orientation and inconsistencies in classification during data processing. This capability enhances our ability to validate the quality of data collection and processing at an early stage, providing guidance to make necessary adjustments to the workflow, to ensure more reliable structural analyses. The integration of Feret signature analysis into standard workflows will allow researchers to adapt their strategies based on real-time data. This adaptability is essential for the rapid progression of projects and for maximizing the efficiency of resources within laboratory and facility settings. 

## Data Availability

The original contributions presented in the study are included in the article, further inquiries can be directed to the corresponding authors.

## Abbreviations

2D	two-dimensional
3D	three-dimensional
Å	Ångström
cryoEM	cryogenic electron microscopy
CTF	contrast transfer function
e^−^	electron
eV	electronvolt
*F_max_*	maximum Feret diameter
kV	kilovolt
SPA	single particle analysis

## References

[1] Kühlbrandt W. (2014). The Resolution Revolution. Science.

[2] Subramaniam S. (2019). The Cryo-EM Revolution: Fueling the next Phase. IUCrJ.

[3] Callaway E. (2020). The Protein-Imaging Technique Taking over Structural Biology. Nature.

[4] Danev R., Yanagisawa H., Kikkawa M. (2019). Cryo-Electron Microscopy Methodology: Current Aspects and Future Directions. Trends Biochem. Sci..

[5] Xu Y., Dang S. (2022). Recent Technical Advances in Sample Preparation for Single-Particle Cryo-EM. Front. Mol. Biosci..

[6] Patel A., Toso D., Litvak A., Nogales E. (2021). Efficient Graphene Oxide Coating Improves Cryo-EM Sample Preparation and Data Collection from Tilted Grids. bioRxiv.

[7] Glaeser R.M., Han B.-G. (2017). Opinion: Hazards Faced by Macromolecules When Confined to Thin Aqueous Films. Biophys. Rep..

[8] Tan Y.Z., Baldwin P.R., Davis J.H., Williamson J.R., Potter C.S., Carragher B., Lyumkis D. (2017). Addressing Preferred Specimen Orientation in Single-Particle Cryo-EM through Tilting. Nat. Methods.

[9] Liu Y.-T., Fan H., Hu J.J., Zhou Z.H. Overcoming the Preferred Orientation Problem in cryoEM with Self-Supervised Deep-Learning. bioRxiv.

[10] Noble A.J., Dandey V.P., Wei H., Brasch J., Chase J., Acharya P., Tan Y.Z., Zhang Z., Kim L.Y., Scapin G. (2018). Routine Single Particle CryoEM Sample and Grid Characterization by Tomography. eLife.

[11] Li B., Zhu D., Shi H., Zhang X. (2021). Effect of Charge on Protein Preferred Orientation at the Air–Water Interface in Cryo-Electron Microscopy. J. Struct. Biol..

[12] Vilela F., Bezault A., Rodriguez De Francisco B., Sauvanet C., Xu X.-P., Swift M.F., Yao Y., Marrasi F.M., Hanein D., Volkmann N. (2022). Characterization of Heterogeneity in Nanodisc Samples Using Feret Signatures. J. Struct. Biol..

[13] Radermacher M., Wagenknecht T., Verschoor A., Frank J. (1987). Three-dimensional Reconstruction from a Single-exposure, Random Conical Tilt Series Applied to the 50S Ribosomal Subunit of *Escherichia coli*. J. Microsc..

[14] Wu M., Lander G.C., Herzik M.A. (2020). Sub-2 Angstrom Resolution Structure Determination Using Single-Particle Cryo-EM at 200 keV. J. Struct. Biol. X.

[15] Xu X.-P., Kim E., Swift M., Smith J.W., Volkmann N., Hanein D. (2016). Three-Dimensional Structures of Full-Length, Membrane-Embedded Human αIIbβ3 Integrin Complexes. Biophys. J..

[16] Mastronarde D.N. (2005). Automated Electron Microscope Tomography Using Robust Prediction of Specimen Movements. J. Struct. Biol..

[17] Punjani A., Rubinstein J.L., Fleet D.J., Brubaker M.A. (2017). cryoSPARC: Algorithms for Rapid Unsupervised Cryo-EM Structure Determination. Nat. Methods.

[18] Volkmann N., Hanein D. (1999). Quantitative Fitting of Atomic Models into Observed Densities Derived by Electron Microscopy. J. Struct. Biol..

[19] Schatz M., Van Heel M. (1990). Invariant Classification of Molecular Views in Electron Micrographs. Ultramicroscopy.

[20] Buades A., Coll B., Morel J.-M. (2005). A Non-Local Algorithm for Image Denoising. Proceedings of the 2005 IEEE Computer Society Conference on Computer Vision and Pattern Recognition (CVPR’05).

[21] Sahoo P., Wilkins C., Yeager J. (1997). Threshold Selection Using Renyi’s Entropy. Pattern Recogn..

